# Sodium and Health: Old Myths and a Controversy Based on Denial

**DOI:** 10.1007/s13668-021-00383-z

**Published:** 2022-02-14

**Authors:** Francesco P. Cappuccio, Norm R. C. Campbell, Feng J. He, Michael F. Jacobson, Graham A. MacGregor, Elliott Antman, Lawrence J. Appel, JoAnne Arcand, Adriana Blanco-Metzler, Nancy R. Cook, Juliet R. Guichon, Mary R. L’Abbè, Daniel T. Lackland, Tim Lang, Rachael M. McLean, Marius Miglinas, Ian Mitchell, Frank M. Sacks, Peter S. Sever, Meir Stampfer, Pasquale Strazzullo, Wayne Sunman, Jacqui Webster, Paul K. Whelton, Walter Willett

**Affiliations:** 1 University of Warwick, W.H.O. Collaborating Centre for Nutrition†, Warwick Medical School, Gibbett Hill Road, CV4 7AL Coventry, UK; 2 University of Calgary, Calgary, Canada; 3 Wolfson Institute of Population Health, Barts and The London School of Medicine & Dentistry, Queen Mary University of London, London, UK; 4 Author, ‘Salt Wars, The Battle Over the Biggest Killer in the American Diet’, Washington, DC USA; 5 Brigham and Women’s Hospital, Harvard Medical School, Boston, USA; 6 Johns Hopkins University, Baltimore, USA; 7 Faculty of Health Sciences, Ontario Tech University, Oshawa, ON Canada; 8 Costa Rican Institute of Research and Teaching in Nutrition and Health, San José, Costa Rica; 9 Temerty Faculty of Medicine, University of Toronto, W.H.O. Collaborating Centre On Nutrition Policy for Chronic Disease Prevention, Toronto, Canada; 10 Medical University of South Carolina, Charleston, USA; 11 Centre for Food Policy, City, University of London, London, UK; 12 Dunedin School of Medicine, University of Otago, Dunedin, New Zealand; 13 Santaros Klinikos Hospital, Vilnius University, Vilnius, Lithuania; 14 Harvard T.H. Chan School of Public Health, Boston, USA; 15 Imperial College School of Medicine, London, UK; 16 Federico II University of Naples, Naples, Italy; 17 Nottingham University Hospitals NHS Trust, Nottingham, UK; 18 The George Institute for Global Health, W.H.O. Collaborating Centre On Salt Reduction†, Sydney, Australia; 19 Department of Epidemiology, Tulane University School of Public Health and Tropical Medicine, New Orleans, USA

**Keywords:** Sodium (salt) intake, Population sodium reduction, Cardiovascular prevention, Public health policy, Ethics, Conflict of interest

## Abstract

***Purpose of Review*:**

The scientific consensus on which global health organizations base public health policies is that high sodium intake increases blood pressure (BP) in a linear fashion contributing to cardiovascular disease (CVD). A moderate reduction in sodium intake to 2000 mg per day helps ensure that BP remains at a healthy level to reduce the burden of CVD.

***Recent Findings*:**

Yet, since as long ago as 1988, and more recently in eight articles published in the *European Heart Journal* in 2020 and 2021, some researchers have propagated a myth that reducing sodium does not consistently reduce CVD but rather that lower sodium might increase the risk of CVD. These claims are not well-founded and support some food and beverage industry’s vested interests in the use of excessive amounts of salt to preserve food, enhance taste, and increase thirst. Nevertheless, some researchers, often with funding from the food industry, continue to publish such claims without addressing the numerous objections. This article analyzes the eight articles as a case study, summarizes misleading claims, their objections, and it offers possible reasons for such claims.

***Summary*:**

Our study calls upon journal editors to ensure that unfounded claims about sodium intake be rigorously challenged by independent reviewers before publication; to avoid editorial writers who have been co-authors with the subject paper’s authors; to require statements of conflict of interest; and to ensure that their pages are used only by those who seek to advance knowledge by engaging in the scientific method and its collegial pursuit. The public interest in the prevention and treatment of disease requires no less.

## Introduction

Sodium intake is a major determinant of blood pressure (BP) [1–3]. A reduction in dietary sodium consumption reduces BP in both individuals and populations [1, 2, 4•]. The effect is dose-dependent; it is detected in both sexes and all ethnic groups, starts in children, becomes greater as we grow older and increases as the baseline BP increases [5–7]. Meta-analyses of randomized controlled trials demonstrate a linear reduction in cardiovascular disease (CVD) when dietary sodium is reduced from 4100 mg/day to 2300 mg/day [8••]. Based on the evidence accrued over the past 40 years, and on repeated, careful, independent scientific reviews conducted by many governmental and non-governmental organizations, national and international public health authorities recommend a reduction in dietary sodium consumption to help prevent and treat hypertension and to help prevent CVD [8••, 9, 10•, 11, 12•, 13•, 14, 15]. The World Health Organization (WHO) [16] and the National Academies of Science, Engineering and Medicine (NASEM) [8••] recommend that dietary sodium intake be less than 2000 and 2300 mg/day, respectively, based on strong to moderate evidence of the impact of sodium on BP and CVD. Such recommendations have been opposed by sectors of the food and beverages industry for decades. High sodium consumption is a source of profit by increasing preference for salty foods, enhancing water binding in meat products to increase weight and therefore price before packaging, and making cheap and unpalatable food edible at minimal cost. High sodium intake also causes thirst and high demand for beverages, including those such as sugar-sweetened beverages manufactured by some of the same industries that produce salty foods [17]. A reduction in BP would reduce the prevalence of hypertension and the use of anti-hypertensive medications, preventing CVD, and reducing costs for the health-care system.

Notwithstanding the compelling evidence, some studies have reported contradictory results on the association between sodium consumption and health outcomes [18–35]. The studies report that, rather than there being a linear rise in CVD as sodium intake rises, CVD declines as sodium levels declines from high levels, with the benefit then leveling off and CVD increasing for lower sodium levels (describing a J-shaped curve). These results cast doubt on the wisdom of global policies recommending a moderate reduction in the consumption of sodium for individuals and populations to help reduce the burden of CVD, which is the leading cause of illness, disability, and death worldwide. The authors of these studies have even suggested that reducing daily sodium consumption below 3000 mg (i.e. 7.5 g of salt) can harm health; this claim has generated controversy [17, 36, 37, 38••, 39–46, 47•, 48–56], often heated debates [37, 44, 57–64], and general confusion for clinicians, health professionals, policy makers, and the public because the results are in stark contrast to the evidence. In some cases, the authors have received financial support from the food and beverages industry, which they have not always declared as a conflict of interest [47•]. Thorough scientific critiques of those publications have consistently raised serious concerns about the quality of the methods used and refuted those conclusions [37, 39, 40, 44, 57, 58, 63, 65–67]. Nevertheless, a small group of scientists continues to publish research based on use of the same flawed methods and without an acknowledgement of the criticisms of their work. This practice of publishing controversial results that are decisively discredited by reputable scientists [68] and scientific authorities [8••] is contrary to the norms of science and the expected behavior of scientists. Moreover, continuing to insist upon the validity of the J-curve representation of data, without recognizing and addressing criticisms and making appropriate amendments [69, 70] reinforces misperceptions about the benefits and risks of reducing sodium consumption (Table 1) [17]. The latest series of controversial publications was published in a single journal [71–78]. As scientists, we share the desire to advance science by using its methods which includes attempting to replicate or reanalyze those studies that arrive at unusual conclusions and to achieve a scientific consensus upon which to make clinical and public health recommendations. Many millions of people’s lives depend upon the quality of such recommendations. Consequently, we wish to use the recent series of publications in the *European Heart Journal* that make controversial claims about sodium’s effect on CVD as a case study to highlight our concerns and to make readers aware of the numerous reasons that these claims are not substantiated.

**Table 1:** Misperceptions about salt reduction: myths and facts

**Myths**	**Facts**
Our body needs sodium	The body efficiently conserves sodium. It is difficult to eat too little sodium as sodium is already in most foods we eat every day. People in some remote areas of the world or in rural areas of developing countries still survive on a fraction of the amount of sodium eaten in the Western world (as low as 100–200 mg per day). Although much table salt is iodized, the required level of iodine can be achieved with sodium intake of 2300 mg/day. There is no evidence of harmful effects of a modest reduction in sodium intake down to 2300 mg per day.
The current sodium intake is a physiologically set normal range in adult humans	During several million years of evolution mankind has survived on very little sodium in the diet (under 1000 mg per day). Even in modern times, this low intake is still seen in the Yanomano and Xingu Indians living in the humid and hot environment of the Amazon jungle. They eat far less than 1200 mg of sodium (3 g of salt) per day, their BP does not rise with age and stroke events are rare. Meanwhile in industrialized populations, the high sodium intake, typically 3000 to 4800 mg of sodium (7.5 to 12 g of salt) per day is recent phenomenon in evolutionary terms. In these groups, BP rises steadily with age, followed by stroke and CHD.
The ‘‘normal’’ sodium intake is between 5.0 and 7.5 g per day (12.5 and 18.8 g salt per day) and a “moderate” intake between 3.0 and 5.0 g per day (7.5 and 12.5 g salt per day)	The range of dietary sodium reported by some as ‘‘normal’’ is only the ‘‘usual’’ range in industrialized westernized countries. It is not a physiological normal. The physiological level compatible with life is seen when access to added dietary sodium is limited, as in parts of Africa, Asia, and South America. Furthermore, this excessive sodium intake is not a matter of personal choice. Only 10–20% of sodium in our diets comes from that added to food by consumers.
Only old people need to worry about how much sodium they eat	Eating too much sodium raises BP at any age, starting at birth and affecting children of all ages. It is best to reduce sodium intake at a young age to form low-salt taste preferences and forestall the onset of hypertension.
Only people with hypertension need to reduce their sodium intake	A reduction in sodium intake reduces BP in both normotensive and hypertensive individuals. It is even more important that people ‘‘without’’ hypertension reduce their sodium intake, because the population-wide number of cardiovascular events that can be attributed to their level of BP is high, but their BP does not make them eligible for drug therapy.
Sodium intake below 3.0 g per day (7.5 g of salt per day) could be potentially harmful	This claim is based on either flawed or unreliable evidence, as extensively argued in recent years (see “Case study: the European Heart Journal” section). On the contrary, there is much evidence that a modest reduction in daily sodium intake (down to 2000 mg) has many beneficial effects on health and is one of the most cost-effective ways to reduce CVD in the population.
Sustained reduction in sodium intake is not feasible in free-living individuals	The experience in the UK (15% or 1.4 g salt per day population reduction achieved in 7 years) and longer in Finland and Japan (about 3 g salt per day population reduction achieved over two decades, though intakes are still excessive) demonstrate that public health policy can lead to substantial reductions in population salt intake. This is paralleled by significant reductions in population BP and in stroke rates, with ensuing cost savings. These salt reductions have very little to do with changing individual behavior, but mainly reflect a healthier environment: the reformulation of industrial-produced and distributed food with lower sodium content. Most individuals in most developed countries have little choice over how much salt they are eating because of the ubiquity of processed food. Secondly, the health benefits of, and progress in achieving, salt reduction are greater if mandatory regulations for food reformulation are introduced.
A reduction in sodium intake below 3.0 g per day activates the renin-angiotensin system	There is no evidence for choosing 3.0 g of sodium per day as a cut-off point. When sodium intake is reduced, the activation of the renin-angiotensin system is a normal physiological response, like that which occurs with diuretic treatment. Outcome trials have demonstrated clear benefits of diuretics on CVD outcomes. Additionally, with a longer-term modest reduction in salt intake, there is only a very small increase in plasma renin activity, and this is true in any ethnic group.
Rock salt, sea salt or other expensive salts are more healthful than table salt	All these salts contain > 95% sodium chloride, whether in grains, crystals, flakes, or with different color appearance.
We need sodium in hot climates or when we exercise because we sweat a lot	We lose only a small amount of sodium through sweat. We are adaptable. The less sodium we eat, the lower the sodium content of our sweat. Thus, in hot climates, it is important to drink plenty of water to avoid dehydration. But we do not need to ingest more sodium.
Consumer taste preferences make change impossible	As sodium intake falls, the taste receptors for sodium in the mouth become more sensitive to lower concentrations within a couple of months. Furthermore, consumer experience in the UK and elsewhere confirms that where sodium has been gradually reduced in major brand products, sometimes concomitant with other reformulations, there has been no reduction in sales and no complaints about taste. Furthermore, once sodium intake is reduced, many people prefer food with less sodium.
Food technology cannot change	The effective UK Food Standards Agency sodium reduction program, as well as other experience, demonstrates that it is possible to remove as much as half of the sodium out from some products gradually without noticeable changes in flavour or consumer acceptance. Finland and Japan have done better still.
Food Safety requires the use of salt	Many companies could reduce sodium significantly in processed meats and other preserved foods. Furthermore, many microbiological modelling tools can be used to help the industry predict the safety and shelf-life of food.

Modified from [17]

## Case Study: the European Heart Journal

Toward the end of 2020 and the beginning of 2021, the *European Heart Journal* published eight articles on sodium and CVD, including one research article [78], one review [76], three commissioned editorials [71, 73, 74], and three commentaries [72, 75, 77]. These articles individually and collectively cast doubt on sodium-reduction recommendations, stating that “there is insufficient evidence to date to recommend a low sodium intake” at the population level [76], and that “it is premature to recommend reducing sodium to low levels if we are […] potentially [to] risk the lives of millions of people worldwide” [74]. By “low” the authors mean sodium intake below 2300 mg/day (5.75 g of salt per day) [76]. Such statements might derail current public health programmes to reduce population dietary sodium consumption to prevent CVD worldwide. It is of particular concern that the evidence offered in these papers to support their recommendation does not reflect the totality of the evidence or rebut the great body of evidence indicating the value of lower-sodium diets. Collectively these articles express opinions based on flawed evidence without due discussion of the scientific criticisms of the methods and evidence that supports reduction in dietary sodium intake globally [1–3, 4•, 6, 7, 8••, 9, 10•, 11, 12•, 13•, 14]. The articles perpetuate old myths about sodium intake, BP, and CVD (Table 1) and create a controversy based on denial of the merits of the existing scientific consensus, with the lack of acknowledgement of the evidence and the unwillingness to directly address the scientific criticisms of their methods [43, 49, 50, 52, 53, 55, 60, 62, 67, 68, 69, 73–75, 79–84].

### How Much Sodium Do We Eat and What are the Sources of Dietary Sodium?

Sodium is an “essential nutrient” in amounts derived from natural food. Above this amount, sodium is added to modern diets through discretionary sources such as salt and monosodium glutamate, and through food processing that leads to consumption of an amount that is more than five times higher than that expected from natural food sources [85]. Studies establishing the physiological requirements for sodium are not available [86]. However, from balance studies and the DASH-sodium trial [87], the 2019 National Academy of Science DRI Report provides an estimate of adequate sodium intake in adults of 1500 mg/day [8••]. In many high-income countries, more than 70% of sodium consumed results from the addition of sodium during food manufacturing, and food preparation in fast-food and sit-down restaurants, with no more than 10–15% of the sodium consumed coming from natural sources, with the remainder resulting from discretionary use in home cooking and at the table [7, 88–90]. In most low- and middle-income countries, however, excessive sodium consumption results from the addition of sodium, high-sodium sauces, and condiments during food preparation, cooking, and at the table [91]. The disparate sources of dietary sodium intake have implications for the choices of population-wide strategies to reduce its consumption. Globalization of the food industry is increasing the exposure of populations in middle- and low-income countries to sodium in processed foods with a transition towards more processed and ultra-processed food consumption [92].

### What Is a “Normal” Sodium Intake?

What we measure today in most human populations is “usual” sodium intake, which cannot be conflated with being biologically “normal.” The Palaeolithic human diet and that of humans living a hunter-gatherer subsistence today contain under 1000 mg of sodium per day [93]. Contemporary hunter-gatherer societies still survive with average sodium intake of 1000 mg per day or considerably less. People in several communities around the world still live with a daily sodium consumption of < 400 mg (< 1 g salt) [94–96], an amount of sodium that is compatible with healthy life. Individuals in these populations have a much lower average BP than is usual in most societies, and their BP does not increase with age. Within a population, sodium (salt) consumption is continuously distributed from low to high [97]. Therefore, definitions of “extremely low, very low, low, normal, high, very high, extremely high”, as used in several articles [25–27, 74, 76, 78, 82, 98] are arbitrary. These concepts, and the consequences of reporting biased interpretation of results, have been extensively reported in the literature, but systematically neglected [74, 76, 80–82]. Therefore, a more standardized nomenclature for the reduction in daily dietary sodium (salt) intake has been suggested, based on evidence (Table 2) [86].

### Does a Reduction in Sodium Intake Reduce Cardiovascular Risk?

Mente et al. [74] argue that there is no “definitive evidence” or any study showing a “clear reduction” in clinical outcomes from reducing sodium intake. The statement is incorrect because there *is* evidence to this effect. The evidence includes randomized clinical trials including TONE [99] and TOHP [100] and meta-analyses of these studies and a few others indicating a 20–30% reduction of cardiovascular events after a period of moderate reduction of sodium intake from 4100 to 2300 mg [2, 8••]. Furthermore, a recent large salt-substitution trial carried out in China showed that a reduction in sodium consumption of 350 mg per day with an increase in potassium consumption of 803 mg caused a statistically significant 14% reduction in fatal and non-fatal strokes over 4.7 years of follow-up, with reductions of nonfatal acute coronary syndrome events (− 30%) and of deaths from any cause (− 12%) [101••], confirming early evidence from a smaller study in Taiwan [102]. While calling for a controlled trial to provide “robust evidence” to support the current global policies, Mente et al. lend their support to an “ecological analysis” of global statistics by Messerli et al. [78]. There are many inherent limitations of such analyses. Messerli et al. [78] correlate sodium and outcomes by country, not by individual. The study design is unable to remove unmeasured confounding (ecological fallacy), a well-known methodological concern that the authors acknowledge and then promptly dismiss. Many countries do not have data on sodium intake and, when available, it is often of poor quality. When comparing “high income” countries (in World Bank Income Class 1), the authors aggregate data from the USA, UK and Canada, Trinidad & Tobago, and Equatorial Guinea. The distribution of wealth in these countries and the ensuing disparities in individual health will have huge effects on life expectancy due to factors other than sodium intake, none of which are accounted for. In addition, Messerli et al. ignore the hard evidence from previous human trials. Yet, Messerli et al. claim their results “argue against dietary sodium intake being a culprit of curtailing life span or being a risk factor for premature death”.

International collaborators of the PURE study and a few others ignore the serious and fundamental flaws of their methods. Such flaws include inaccurate dietary assessment tools [18, 22] and spot urine samples with discredited conversion formulas to estimate 24-h urinary sodium excretion [20, 23, 25, 27–29, 32, 34, 35]. In large epidemiological studies, collection of spot urines is feasible but is chosen at the expense of validity when such data are used to predict risk of clinical outcomes [41, 103–105]. The use of sodium concentrations in fasting spot samples extrapolated to 24 h urinary sodium excretion using the Kawasaki or other formulas is an inappropriate method for estimating salt intake in individuals [106–108]. The authors’ validation study [109] criticized at the time of its publication [110], denies the presence of a significant bias when estimating individuals’ sodium excretion as shown in the Bland–Altman plots. However, the results of other validation studies are not in agreement [106]. They also fail to mention that a similar validation study in the Chinese cohort of the PURE study (the largest sample in the PURE study) showed up to 7000 mg/day differences between estimated and measured 24-h urine sodium, as well as low correlations and high systematic bias in Bland–Altman plots. The validation study concluded: “a more accurate method is needed to estimate 24-h urine sodium from spot samples …” [111]. The authors insist on the concept that the method could be useful to assess population means. However, they use data on individuals when assessing risk prediction in a cohort study design [25]. This is misleading because it has been long established that several 24-h urine collections are needed to approximate an individual’s usual sodium intake with a high degree of confidence (i.e. within 10%) and without bias [112–115]. Furthermore, the formulas themselves, independent of sodium, are important contributors to the J-shaped association between sodium intake and CVD or mortality, because the formulas make use of age, sex, urinary creatinine concentration, height, weight, most of which are independent predictors of CVD and mortality [116••, 117••]. By contrast, most cohort studies that used the method of repeated 24-h urine collections to assess salt intake, identified beyond doubt a graded, mostly linear, relationship between sodium excretion and cardiovascular outcomes with no increase in CVD risk at lower sodium intakes [66, 117••, 118, 119].

The potential for reverse causality is another problem affecting many of the studies reporting a J-shaped association between sodium and outcomes [23, 29–31, 66, 118]. The same research group in one of its reports presents a pooled analysis of four studies, namely the PURE and EpiDREAM, both population-based observational studies, and two observational analyses based on the non-randomized data bases of both ONTARGET and TRANSCEND clinical trials [28]. An important flaw is the consistent use of sick populations and patient groups to study the implications of a moderate reduction in sodium consumption in the general population. The combined sample from ONTARGET and TRANSCEND study included 28,800 participants from high-risk patients to undergo randomized clinical trials of anti-hypertensive treatments. Those studied were old (mean age 66.5 ± 7.2 years; 2.4 years older in the lower compared to the higher sodium intake group), 71% were men (but the lower sodium group included 54% women), all with significant previous disease (48% with MIs, 21% CVAs, 70% hypertension and 37% diabetes), all highly medicated with beta-blockers (57%), diuretics (29%), calcium channel blockers (35%), and ~ 75% on blockers of the renin-angiotensin system. The proportion of patients on diuretics was high in both the lower (41%) and the higher (43%) sodium intake groups [28]. The reported higher cardiovascular mortality in the lower sodium group was, in fact, only detected in the composite outcome of total CV death. This was exclusively accounted for by excess heart failure in this group, but not excess MI, stroke or non-CV death. Taken together, the results suggest that the patients at high risk of heart failure in the lower sodium intake group were more likely to take diuretics and be at higher risk of death due to the high mortality detected in that group (reverse causality) [37, 44, 57, 120]. In other words, the groups were not representative of the general population and confounders related to pre-existing conditions ought to have been addressed in the report. Similar attention should be given to the PURE Study, an on-going epidemiological cohort study that has enrolled over 156,000 individuals in 17 countries. The paper reporting the results on sodium intake, BP and CVD analyzed only 65% of the original cohort (102,000 out of 156,000 participants) who were able to provide a spot urine sample. Compared to the overall original cohort, the sodium cohort had fewer participants from India (5 vs 18%) and more from China (42% vs 30%), with an imbalanced distribution across sodium groups (27). The lower-sodium group was 2.8 years older, had fewer men (29.6 vs 58.1%), fewer participants from Asian ancestry (33.8 vs 73.0%), more with history of CVD (9.2 vs 7.1%) and diabetes (10.6 vs 8.4%), and a greater proportion of people on regular medications, suggesting the presence of self-selected sicker participants in the lower-sodium group. These imbalances can result in confounding if not properly controlled and suggest that there may be additional unmeasured confounders, including energy intake and physical activity, both of which are poorly measured in epidemiological studies Furthermore, the use of invalid methods to assess sodium intake introduces a bias [11, 41, 121–123]. Studies with more stringent quality control features have been able to avoid such biases and have obtained more reliable results [118]. The EpiDREAM cohort screened people at high-risk for incident type 2 diabetes, the majority being of non-European ethnicity, and over 70% being obese women, with a high proportion taking medications [124]. None of these four studies’ results can be generalized to inform current public health policies for a moderate reduction in sodium consumption in populations. The 2019 NASEM Report viewed these studies as particularly biased, with the J-shaped curves likely due to methodological limitations [8••].

The flaws, reproduced in all countries of the PURE Study, are responsible for the artifactual J-shaped curve for the association between urinary sodium and clinical outcomes [116••]. A graded reduction in CVD (without a J-shape curve) has been described in meta-analyses of randomized controlled trials across the same levels of dietary sodium where the PURE and other controversial cohort studies find increasing CVD for lower sodium levels [8••] A J-curve has not been seen in meta-analyses of cohort studies that have employed high quality methods likely to avoid spurious paradoxical results [8••, 66, 118, 119, 125]. Twenty-four hour urine samples are the tool recommended by many regions of the World Health Organization to assess population sodium consumption [126–129]. However, the WHO STEPS survey still allows spot urines [130], despite of the evidence that the measures are flawed. Spot urines may be unable to monitor effectively changes in average population sodium consumption over time, an important indicator of the effectiveness of sodium-reduction policies [13•, 131, 132].

### Mis-reporting Evidence and Denial

**Table 2:** Proposed nomenclature for sodium (salt) intake and the reductions in dietary sodium (salt)

**Terminology**	**Sodium (mg per day)**	**Salt (g per day)**
**Intake**		
Normal (physiological)	< 1000	< 2.5
Recommended	≲2000	≤ 5.0
High	≥ 2000	≥ 5.0
Very high	> 4000– ≤ 6000	> 10– ≤ 15
Extremely high	> 6000	> 15
**Reduction**		
Small	< 1000	< 2.5
Moderate	1000–2000	2.5–5.0
Large	> 2000	> 5.0

Modified from [86]

Both the study by Messerli et al. [78] and the accompanying editorial by Mente et al. [74] claim that one strength of Messerli’s analysis is that “sodium intake was estimated from 24-h urine collections”. A close perusal of the data source for the 24-h urinary sodium estimates used in the Messerli et al. report [133] indicates that this statement is incorrect and misleading. The Powles et al. study from which Messerli et al. obtained their 24-h urinary sodium estimates used a combination of 142 urine-based and 103 diet-based estimates. Several imputations were then made from 79 datapoints from 26 surveys where both urine and diet estimates were available. Imputations of average salt consumptions were then used for countries that had no surveys. In other words, sodium intake was not estimated from 24-h urine collections in Messerli’s analysis.

Moreover, in their editorial, Mente et al. argue that “it is premature to recommend reducing sodium to *low levels* [< 3 g of sodium or < 7.5 g of salt per day in the authors’ arbitrary classification] if we are to avoid a large waste of resources” [74]. Extensive health economic analyses across the world estimate that population salt reduction is one of the most cost-effective (and in some settings cost-saving) public health strategies to prevent cardiovascular disease globally [102, 134–160], and this policy has been adopted by the World Health Organization as one of the “best-buys” to help prevent CVD [161].

## Reflections and Conclusions

The articles recently published in the *European Heart Journal* are based on flawed, biased, incomplete, and inaccurate science. In addition, the level of misrepresentation and denial of the enormous body of evidence supporting recommendations to reduce dietary sodium intake raises serious concerns. A false sense of equipoise now obfuscates the facts and creates an aura of controversy that adds credibility to dissenting scientists who publish in high-impact journals. Their science is affected by poor rigour in research methodology, consistent bias and misrepresentation of the entire body of evidence available. The overrepresentation of dissenting paradoxical viewpoints in scientific journals, conferences, media, blogs, and other information outlets has “…succeeded in creating a false equivalence, even when there is only one credible side”, as an observer said [162].

The resurgence of advocacy against reducing dietary sodium intake might have occurred for complex reasons: conflict of interest and commercial bias have been a long-standing issue, with some individuals known to be consultants to the salt, food and pharmaceutical industries. Effort that creates a “debate” in the scientific literature when there is no authentic debate can generate research funding. Many reasons have hampered the ability to refute the false and misleading claims. They often include lack of public access to the data allegedly supporting research claims, unscientific conduct, and unclear rules as to which institution is responsible for policing ethics obligations when many institutions are involved (granting bodies, research ethics committees, journals, health and scientific organizations, and governments) [38••]. Finally, controversial scientific papers might be accepted for publication because they are more “interesting” and journals might apply lower standards regarding their methodological rigour and reproducibility [163].

For the case study presented, there has been a lapse in implementing the *European Heart Journal* “Conflict of Interest Policy”, which raises questions about the scientific publishing enterprise. Editorial writers [74] have been co-authors [76] with authors of a paper they commented on, as with a recent paper [78]. This could be “perceived” as a conflict of interest, especially when glaring omissions are detected in the editorial. Furthermore, the article by O’Donnell et al. [76], rather than presented as a View Point or Debate, was portrayed by the journal as a Clinical Review (listed in the Instructions for Authors as State-of-the-Art Review), thus misrepresenting the field. Conflicts of interest were not declared, thus undermining public trust in the scientific process and the credibility of the published articles [38••]. In nutrition science, there has been a long-standing lack of ethical guidance and relaxed implementation from all stakeholders [164]. Journals and editors are responsible for the scientific integrity of what they publish [165]. The studies we have reviewed on salt and CVD indicate the need to revamp the current medical publishing system [166, 167]. The present case study has identified issues of significant societal consequence that are critical to address to maintain public trust in the scientific process. We have identified numerous challenges to scientific integrity that plague science (like those seen in the past regarding tobacco and currently regarding climate change). The case study highlights the need to develop, implement and enforce higher research quality and publishing standards to safeguard public policy in areas of nutrition where millions of lives are at risk.

Evidence supporting population-wide reduction in sodium intake is consistent, robust, and endorsed by such major health authorities as the WHO [16] and NASEM [8••]. A comprehensive public health approach to reduce sodium in the food supply is underway to prevent millions of unnecessary deaths and billions in health-care costs. This important work aims literally to save lives. It should not be impeded or derailed by fatally flawed research [168].
